# Supplementary material for: Morphological diversity in tenrecs (Afrosoricida, Tenrecidae): comparing tenrec skull diversity to their closest relatives
Source: PeerJ. 2015 Apr 30;3:e927. doi: 10.7717/peerj.927 (PMC4419542; doi:10.7717/peerj.927)
Supplement: Supplemental Information 1 [file peerj-03-927-s001.pdf]

Supplementary Material

Landmark descriptions

The information below provides further details about the landmarks we used to summarise morphological shape in tenrec and golden mole skulls. Landmark numbers and curve descriptions refer to Figure 2 in the main paper (example of a tenrec skull) and also Figure S1 (example golden mole skull). One of us (SF) placed all of the landmarks on each picture.

Skulls: dorsal view

Most of our landmarks in this view are relative (type 3) points which represent overall morphological shape but not necessarily homologous biological features (Zelditch et al., 2012). We placed ten landmarks and drew four semilandmark curves to represent the shape of both the braincase (posterior) and nasal (anterior) area of the skulls. Table S1 describes how we defined the landmarks and outline curves for our images of skulls in dorsal view.

Table S1: Descriptions of the landmarks (points) and curves (semilandmarks) for the skulls in dorsal view

Landmark	Description
1 + 2	Left (1) and right (2) anterior points of the premaxilla
3	Anterior of the nasal bones in the midline
4 + 5	Maximum width of the palate (maxillary) on the left (4) and right (5)
6	Midline intersection between nasal and frontal bones
7 + 8	Widest point of the skull on the left (7) and right (8)
9	Posterior of the skull in the midline
10	Posterior intersection between saggital and parietal sutures
Curve A (12 points)	Outline of the braincase on the left side, between landmarks 7 and 9 (does not include visible features from the lower (ventral) side of the skull)
Curve B (10 points)	Outline of the palate on the left side, between landmarks 1 and 4 (outline of the rostrum only, not the shape of the teeth)
Curve C (12 points)	Outline of the braincase on the right side, between landmarks 8 and 9 (does not include visible features from the lower (ventral) side of the skull)
Curve D (10 points)	Outline of the palate on the right side, between landmarks 2 and 5 (outline of the rostrum only, not the shape of the teeth)

Skulls: ventral view

Most of the landmarks in this view are concentrated around the dentition and palate of the animals. We identified 13 landmarks and drew one outline curve (resampled to 60

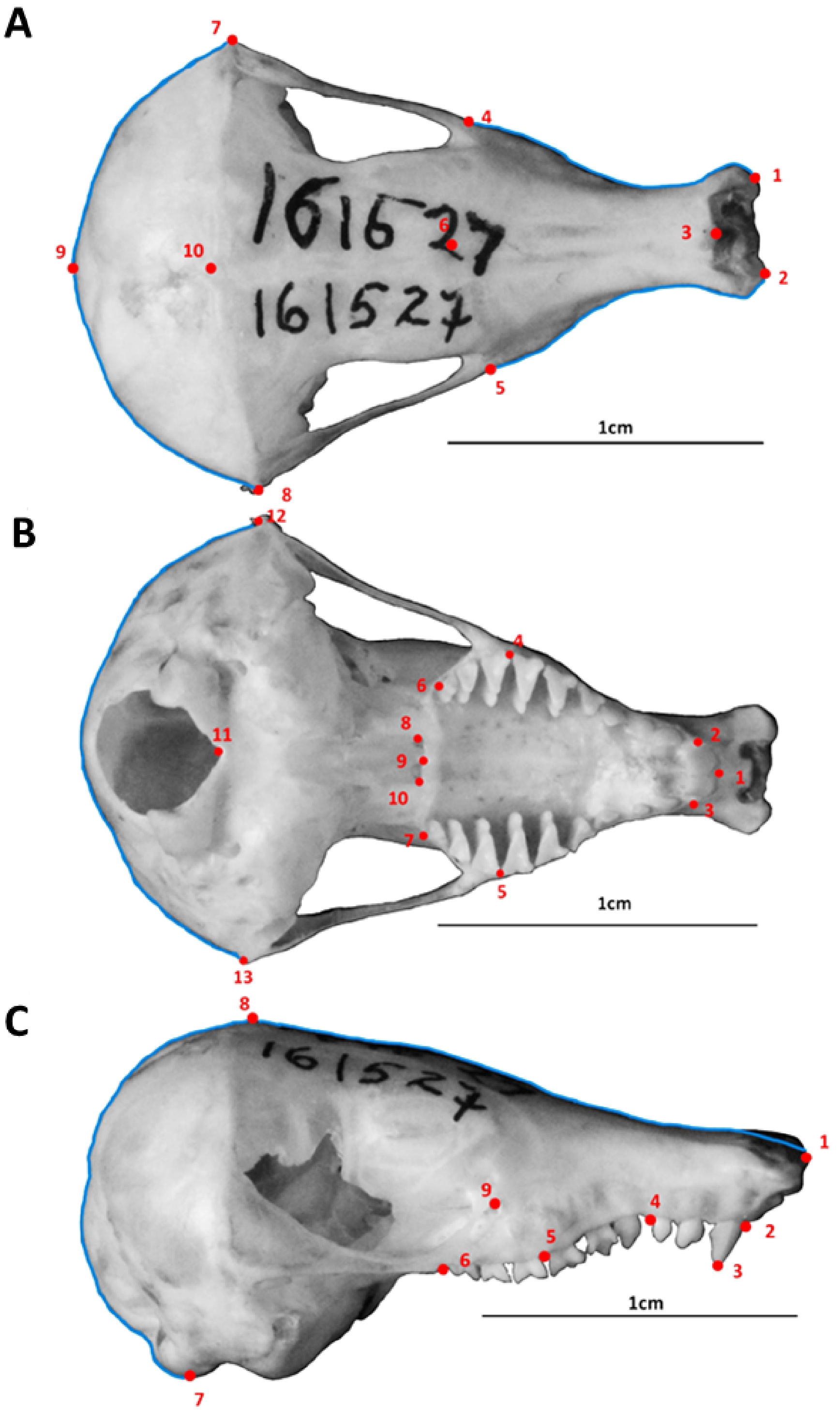

Figure S1: Landmarks (numbered points) and curves (outlines) for the skulls in dorsal (A), ventral (B) and lateral (C) view. See Tables S1, S2 and S3 for more detailed landmark descriptions. The skulls are an example of a *Chlorotalpa duthieae* (Duthie's golden mole), museum accession number AMNH 161527.

semilandmark points) around the back of the skull between landmarks 12 and 13. The high variability of our species' basi-cranial regions and difficulties associated with identifying developmentally or functionally homologous points precluded designation of additional landmarks towards the back of the skulls. Table S2 outlines the descriptions of the landmarks we used for the ventral skull photos.

Table S2: Descriptions of the landmarks (points) and curves (semilandmarks) for the skulls in ventral view.

**Landmark**	**Description**
1	Anterior point of the palate
2 + 3	Posterior, lateral extremity of the right (2) and left (3) incisor
4 + 5	Anterior, outer point of the first molar on the right (4) and left (5)
6 + 7	Posterior, outermost point of the last molar surface on the right (6) and left (7)
8	Widest point of the curve of the palatine on the right side
9	Posterior point of the palatine in the midline
10	Widest point of the curve of the palatine on the left side
11	Anterior of the occipital foramen in the midline
12 + 13	Widest (extreme lateral) point of the braincase on the right (12) and left (13)
Curve*	Outline of the back of the skull (between landmarks 12 and 13), 60 points

*This curve does not necessarily trace homologous features because of the variation in the position of the foramen magnum.

Skulls: lateral view

We placed nine landmarks on the photographs of skulls in lateral view and also drew two semilandmark curves to represent the shape of the back of the skull (between landmarks 7 and 8, re-sampled to 20 semilandmark points) and the top of the skull (between landmarks 8 and 1, re-sampled to 15 semilandmark points). Table S3 describes our definitions for each of the landmark points. If specimens were damaged on their right side we reflected photographs of the left lateral side of the skull so that all the images were in the same orientation.

Table S3: Descriptions of the landmarks (points) and curves (semilandmarks) for the skulls in lateral view.

**Landmark**	**Description**
1	Anterior, upper tip of the nasal bone
2	Anterior of the alveolus of the first incisor
3	Lowest point of the first incisor
4	Posterior of the alveolus of the last incisor
5	Anterior tip of the alveolus of the first molar
6	Posterior tip of the alveolus of the last molar
7	Lowest point of the basi-occipital (base of the back of the skull)
8	Highest point of the braincase
9	Highest point of the infraorbital foramen
Curve A	Between points 7 and 8
(20 points)	Back of the skull from the lowest to highest points
Curve B	Between points 8 and 1
(15 points)	From the highest point of the braincase to the front of the nasal

Potential morphometrics errors

While 2D methods are an accepted means of comparing morphological shape (e.g. Adams et al., 2004; Mitteroecker and Gunz, 2009), particularly for comparing skull morphologies of small mammals (e.g. Cardini, 2003; Panchetti et al., 2008; White and Searle, 2008; Barrow and Macleod, 2008; Scalici and Panchetti, 2011), the inherent discrepancies associated with comparing three dimensional objects using two dimensional pictures can introduce some potential problems of possible image distortion (Arnqvist and Mårtensson, 1998). Similarly, human error with how landmarks are positioned on specimens could also introduce noise into further analyses. In contrast to detailed intraspecific work (e.g. Bornholdt et al., 2008; Blagojević and Milošević-Zlatanović, 2011), photographic or landmark placement errors are unlikely to be significant in our interspecific study since one would expect that the morphological variation among species is large enough to be detected as a signal above any background noise associated with methodological error (Arnqvist and Mårtensson, 1998). Nevertheless, it is still important to assess measurement error in a morphometric data set to increase confidence in the

outcome of final analyses. We identified two possible sources of morphometric measurement error: specimen orientation and placement of landmarks.

Variation in the orientation of specimens for photography is one of the main sources of error in 2D morphometric studies (Adriaens, 2007). If specimens are not placed on a flat plane or in a consistent position relative to the camera, areas of the object which are tilted towards the camera will appear to be larger than in reality, distorting any subsequent morphometric analyses of the shape. One of us (SF) placed the landmarks on each set of pictures so inter-observer variation in landmark placement is not an issue for our study. However, repeatability and reliability of our choice of landmarks could affect the final results of the analyses (Arnqvist and Mårtensson, 1998).

To measure potential orientation error, we photographed the skulls (dorsal, ventral and lateral views) of each specimen three times, cycling through the photos so that the specimen was removed and re-positioned before every shot (Viscosi and Cortini, 2011). We used a subset of the ventral skull images to test for two sources of error: specimen orientation and landmark placement error (Arnqvist and Mårtensson, 1998; Barrow and Macleod, 2008). Of the specimens which we photographed three times, we chose a random subset of seven skulls from four different Families: three tenrecs and single representatives of shrews, moles, hedgehogs and golden moles. We copied these images and placed landmarks on three copies of each image to compare variation in landmark placement within each orientation. This gave nine replicates of each skull: three separate photos, and three copies of each of those photos. As before, we ran a general Procrustes alignment (Rohlf and Marcus, 1993) of the specimens, calculated the average shape values for each skull (average of all nine pictures) and used these values for a principal components (PC) analysis.

We used a linear mixed effects model to model the shape variation (first PC axis) associated with specimen (fixed effect) and two nested random effects: photo identity nested within specimen and image replicate nested within each photograph. This nested approach was necessary to deal with the hierarchical nature of our data subset: three photos of every specimen and three replicates of each of those images. We ran the test using the lmer function in the lme4 package (Bates et al., 2014).

We found that specimen orientation and landmark placement have negligible effects on the overall shape variation between different skulls: the random effect variables of photo identify and image replicate explained less than 0.0001 % of the overall shape variation among different skulls. Therefore, we are confident that shape variation among the specimens in the rest of the analyses reflects true morphological differences rather than methodological error.

Sample size

The total number of photographs used for each analysis differed slightly because some skull specimens were damaged and could only be used for certain views (see main text). Table S4 summarises the total number of specimens used for each of the three analyses. We accounted for differences in sample size by analysing the average skull shape of each species and using permutation tests to ensure that differences in relative morphological diversity were not artefacts of differences in overall group size (see main text).

Table S4: Summary of the sample size (number of skull specimens) used for each analysis: skulls in dorsal, ventral and lateral view.

Family	Species	Number of Specimens		
		Dorsal	Ventral	Lateral
Chrysochloridae	*Amblysomus corriae*	1	1	1
Chrysochloridae	*Amblysomus hottentotus*	4	4	4
Chrysochloridae	*Calcochloris leucorhinus*	3	3	3
Chrysochloridae	*Calcochloris obtusirostris*	4	3	2
Chrysochloridae	*Carpitalpa arendsi*	1	1	1
Chrysochloridae	*Chlorotalpa duthieae*	3	2	3
Chrysochloridae	*Chrysochloris asiatica*	4	4	3
Chrysochloridae	*Chrysochloris stuhlmanni*	4	4	4
Chrysochloridae	*Chrysospalax trevelyani*	4	4	4
Chrysochloridae	*Chrysospalax villosus*	1	1	1
Chrysochloridae	*Cryptochloris wintoni*	2	2	2
Chrysochloridae	*Eremitalpa granti*	3	3	3
Tenrecidae	*Echinops telfairi*	5	5	5
Tenrecidae	*Geogale aurita*	5	4	5
Tenrecidae	*Hemicentetes nigriceps*	5	4	4
Tenrecidae	*Hemicentetes semispinosus*	6	5	5
Tenrecidae	*Limnogale mergulus*	1	1	1
Tenrecidae	*Microgale brevicaudata*	5	5	5
Tenrecidae	*Microgale cowani*	6	6	6
Tenrecidae	*Microgale dobsoni*	5	4	5
Tenrecidae	*Microgale drouhardi*	5	5	5
Tenrecidae	*Microgale dryas*	5	5	4
Tenrecidae	*Microgale fotsifotsy*	5	5	5
Tenrecidae	*Microgale gracilis*	5	5	4
Tenrecidae	*Microgale grandidieri*	5	5	5
Tenrecidae	*Microgale gymnorhyncha*	5	5	5
Tenrecidae	*Microgale jobihely*	5	5	5
Tenrecidae	*Microgale longicaudata*	5	5	5
Tenrecidae	*Microgale monticola*	5	5	5
Tenrecidae	*Microgale parvula*	4	4	5
Tenrecidae	*Microgale principula*	5	5	5
Tenrecidae	*Microgale pusilla*	5	5	5
Tenrecidae	*Microgale soricoides*	5	4	4
Tenrecidae	*Microgale taiva*	5	5	4
Tenrecidae	*Microgale talazaci*	5	5	5
Tenrecidae	*Microgale thomasi*	5	5	5
Tenrecidae	*Micropotamogale lamottei*	2	2	2
Tenrecidae	*Micropotamogale ruwenzorii*	1	1	1
Tenrecidae	*Oryzorictes hova*	5	5	5
Tenrecidae	*Oryzorictes tetradactylus*	6	6	6
Tenrecidae	*Potamogale velox*	6	6	5
Tenrecidae	*Setifer setosus*	5	4	3
Tenrecidae	*Tenrec ecaudatus*	6	5	6

References

Adams, D. C., F. J. Rohlf, and D. Slice. 2004. Geometric morphometrics: Ten years of progress following the "revolution". Italian Journal of Zoology 71:5–16.

Adriaens, D. 2007. Protocol for error testing in landmark based geometric morphometrics. `http://www.fun-morph.ugent.be/Miscel/Methodology/Morphometrics.pdf`.

Arnqvist, G. and T. Mårtensson. 1998. Measurement error in geometric morphometrics; empirical strategies to assess and reduce its impact on measures of shape. Acta Zoologica Academiae Scientiarum Hungaricae 44:73–96.

Barrow, E. and N. Macleod. 2008. Shape variation in the mole dentary (Talpidae: Mammalia). Zoological Journal of the Linnean Society 153:187–211.

Bates, D., M. Maechler, B. Bolker, and S. Walker. 2014. lme4: Linear mixed-effects models using Eigen and S4.

Blagojević, M. and S. Milošević-Zlatanović. 2011. Sexual shape dimorphism in Serbian roe deer (*Capreolus capreolus* L.). Mammalian Biology - Zeitschrift für Säugetierkunde 76:735–740.

Bornholdt, R., L. R. Oliveira, and M. E. Fabián. 2008. Size and shape variability in the skull of *Myotis nigricans* (schinz, 1821) (chiroptera: Vespertilionidae) from two geographic areas in brazil. Brazilian Journal of Biology 68:623–631.

Cardini, A. 2003. The geometry of the marmot (Rodentia: Sciuridae) mandible: phylogeny and patterns of morphological evolution. Systematic Biology 52:186–205.

Mitteroecker, P. and P. Gunz. 2009. Advances in geometric morphometrics. Evolutionary Biology 36:235–247.

Panchetti, F., M. Scalici, G. Carpaneto, and G. Gibertini. 2008. Shape and size variations in the cranium of elephant-shrews: a morphometric contribution to a phylogenetic debate. Zoomorphology 127:69–82.

Rohlf, J. and L. Marcus. 1993. A revolution in morphometrics. Trends in Ecology and Evolution 8:129–132.

Scalici, M. and F. Panchetti. 2011. Morphological cranial diversity contributes to phylogeny in soft-furred sengis (Afrotheria, Macroscelidea). Zoology 114:85–94.

Viscosi, V. and A. Cortini. 2011. Leaf morphology, taxonomy and geometric morphometrics: a simplified protocol for beginners. PLoS ONE 6:e25630.

White, T. and J. Searle. 2008. Mandible asymmetry and genetic diversity in island populations of the common shrew, *Sorex araneus*. Journal of Evolutionary Biology 21:636–641.

Zelditch, M., D. Swiderski, and D. Sheets. 2012. Geometric Morphometrics for Biologists, 2nd edition. Academic Press, Elsevier.
